# The prevalence of adult-onset isolated dystonia in Finland 2007-2016

**DOI:** 10.1371/journal.pone.0207729

**Published:** 2018-11-20

**Authors:** Rebekka Ortiz, Filip Scheperjans, Tuomas Mertsalmi, Eero Pekkonen

**Affiliations:** Department of Neurology, Helsinki University Hospital and Department of Clinical Neurosciences (Neurology), University of Helsinki, Helsinki, Finland; Universidade de Sao Paulo Faculdade de Medicina, BRAZIL

## Abstract

**Background:**

Dystonia is a group of chronic diseases, causing considerable physical and psychosocial stress to patients and health care expenses. We studied the prevalence of different dystonia types in Finland in the years 2007–2016.

**Methods:**

All patients with an ICD-10 code of dystonia were retrieved from the national care register. Average age-adjusted yearly prevalence was assessed for adult-onset isolated idiopathic or hereditary dystonia types from patient records from the Uusimaa and Pirkanmaa provinces.

**Results:**

1316 patients were confirmed to have adult-onset isolated idiopathic or hereditary dystonia based on hospital records from two provinces. On average, the age-adjusted prevalence for all adult-onset dystonia was 405 per million and for cervical dystonia 304 per million. For other dystonia types the prevalence ranged from 1–33 per million.

**Conclusions:**

Adult onset cervical dystonia was the most common type of dystonia with relatively high prevalence in Finland compared with other countries. The prevalence of other types of dystonia was similar compared with other European studies. The higher prevalence of cervical dystonia may be partially explained by the better coverage of patients in public health care, but genetic and exogenous factors might contribute to it.

## Introduction

Dystonia is a group of chronic diseases characterized by abnormal postures and movements created by involuntary muscle contractions [1]. Although some gene mutations have been linked to dystonia and 25% of focal dystonia patients have an affected family member [2], the etiology is in most cases unknown.

Dystonia can be categorized by type to focal, segmental, multifocal, generalized and hemidystonia, and by affected body region to upper cranial, lower cranial, laryngeal, cervical, upper limb, lower limb, and axial dystonia. Dystonia can be further divided by age of onset and the diurnal variation of symptoms. The early-onset dystonia more often progress to generalized type and are of hereditary or acquired origin, whereas adult-onset dystonia is more often idiopathic and focal type dystonia. [1]

Dystonia is the third most common movement disorder and even though it does not reduce life expectancy, it causes considerable physical and psychosocial stress to patients [3]. In general, the prevalence of dystonia has been reported to be around 164 per million, focal cervical dystonia (CD) being the most common type of dystonia [4]. In southwestern Finland, the dystonia prevalence was studied in 2000 as part of a larger European study. However, the number of patients was small and the patient material was partly collected through media [5]. Besides CD, the country-specific prevalence values were not available [5]. Dystonia is a long-term disease, causing disability and health care expenses. Moreover, deep brain stimulation has emerged as important second-line treatment for severe drug resistant dystonia. Thus, it is important to assess the current prevalence of dystonia.

We studied the prevalence of adult onset isolated idiopathic and isolated hereditary dystonia types in Pirkanmaa and Uusimaa regions (average population in 2007–2016 was 2 043 819, and of these over 20 years old 1 580 758) of southern Finland based on national care register data and patient records.

## Materials and methods

Patients with an ICD-10 diagnosis of dystonia (G24 dystonia, G24.1 idiopathic familial dystonia, G24.2 idiopathic non-familial dystonia, G24.3 spasmodic torticollis, G24.4 idiopathic orofacial dystonia, G24.5 blepharospasm, G24.8 other dystonia, G24.9 dystonia, unspecified) in the years 2007–2016 were retrieved from the national care register of the National Institute of Health and Welfare. The ICD-10 classification has been used in Finland since 1996 and care register includes the diagnoses of all visits in special health care services. Patients could have diagnosed with more than one dystonia type.

Patient records for validation were available from the University hospitals of Helsinki and Tampere, covering the vast majority of the dystonia patients in the Uusimaa and Pirkanmaa provinces. The patient records were screened by R.O. for the exact dystonia diagnosis according to classification of dystonia [1]. The reliability of ICD-10 dystonia diagnoses was checked by verifying the dystonia diagnosis in all available patient records including onset in all age groups and non-isolated or acquired dystonia.

Dystonia was classified as focal, segmental, multifocal, generalized or hemidystonia. Dystonia was defined as followed: focal, if only one body region was affected; segmental, if two or more contiguous body regions were affected; multifocal, if at least two non-contiguous body regions were affected; generalized, if trunk and at least two other body regions were affected; hemidystonia, if several body regions were affected and restricted to one side of the body. Focal dystonia was further classified by body distribution to upper cranial, lower cranial, laryngeal, cervical, upper limb, lower limb or axial dystonia. The type of dystonia was changed accordingly, if spreading or other change in dystonia type occurred. The information for dystonia etiology diagnosis, and age of onset was also obtained from patient records. The age of diagnosis day was determined as first patient record day when diagnosis was set. Adult-onset was defined as dystonia onset over 20 years of age. Possible diurnal fluctuations were acknowledged.

Dystonia was considered isolated if dystonia was the sole neurological manifestation. However, the possible occurrence of tremor did not exclude the diagnosis of isolated dystonia. The etiology was classified as idiopathic, hereditary or acquired. The patients with acquired dystonia due to known cause, including tardive dystonia, were removed from analysis. The degree of heredity could not be analyzed accurately enough from patient records and was not analyzed further. Patients whose dystonia were not adult-onset isolated and idiopathic or hereditary were excluded from further analysis. If not otherwise stated, in the following text the term dystonia refers to adult-onset isolated dystonia of idiopathic or hereditary origin.

For studied dystonia classes in Uusimaa and Pirkanmaa provinces, the yearly prevalence was counted from the index day to last visit with dystonia diagnosis separately every year 2007–2016. The number of patients was compared to the yearly population size retrieved from Statistics Finland [6]. For age-specific rates, the age groups were divided into four groups: 20–39 years, 40–59 years, 60–79 years and over 80 years. Average prevalence with standard deviation was counted from the yearly prevalence of the years 2007–2016. Direct age standardization of crude prevalence was done using the 2013 European Standard population [7]. Mann-Whitney U -test was used to compare the age of diagnosis between different dystonia types, and p < 0.05 was considered statistically significant. The statistical analysis was done using SPSS version 24.0 (SPSS Inc., Chicago, IL, USA).

## Results

**Fig 1:**
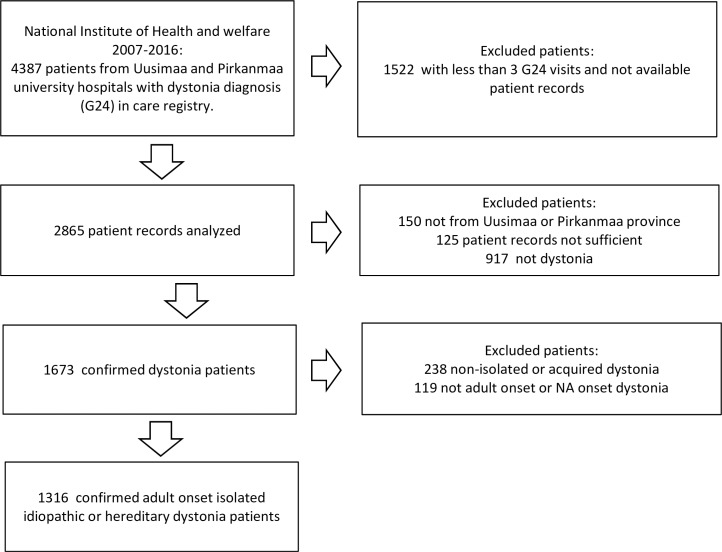
The flow chart of number of included and excluded patients.

Of 4387 patients from the Uusimaa and Pirkanmaa provinces, patients with less than three visits and no patient records in Tampere or Helsinki university hospitals were removed from further analysis. Hence, 2865 patients from these provinces were included for further analysis. Analysis of the available patient records confirmed adult onset isolated idiopathic / hereditary dystonia in 1316 patients (Fig 1). The dystonia diagnosis was done mostly by neurologists, but the diagnosis for laryngeal dystonia was in some cases made by phoniatrician and the diagnosis for blepharospasm (upper cranial dystonia) by ophthalmologists. Of all available patient records with ICD-10 dystonia diagnosis, 17% of the patients with less than 3 dystonia diagnosis visits eventually had dystonia according to medical records (135 of 802 patients) (S1 Fig).

The combined demographic data and average prevalence in different dystonia types altogether, and in different age groups in these provinces are summarized in Table 1. No major differences were observed between these two provinces. Age-adjusted prevalence of all dystonia patients was on average 405 per million. Of these, focal CD had the highest prevalence (304 per million). The prevalence of focal upper cranial (mostly blepharospasm), laryngeal and upper limb dystonia varied between 12 and 26 per million. Clear female predominance was seen in all dystonia types with male to female (M:F) ratio of 1:1,4–1:13, except for focal upper limb dystonia, where M:F ratio was 1:1,1 (Table 1). The M:F-relationship was not counted for dystonia types with less than ten patients.

**Table 1:** The demographic data, average dystonia age-adjusted prevalence and crude prevalence in different age groups 2007–2016.

	Prevalence			Crude prevalence per million ± SD in different age groups			
Dystonia type	per million ± SD (n)	M:F	Age at diagnosis day (n)	20–39 years	40–59 years	60–79 years	over 80 years
All dystonia	405 ± 46 (781)	1:2,7	54 ± 12 (1115)	91 ± 6	650 ± 37	889 ± 146	415 ± 83
Focal cervical	304 ± 34 (589)	1:2,9	53 ± 11 (793)	64 ± 3	527 ± 31	641 ± 109	220 ± 49
Focal upper cranial	26 ± 5 (47)	1:2,7	64 ± 13 (78)	2 ± 2	14 ± 4	74 ± 8	138 ± 39
Focal upper limb	15 ± 7 (31)	1:1,1	51 ± 13 (81)	11 ± 2	31 ± 14	19 ± 10	2 ± 5
Focal laryngeal	12 ± 5 (22)	1:2,1	59 ± 17 (42)	4 ± 2	14 ± 5	23 ± 9	34 ± 18
Focal lower cranial	5 ± 2 (9)	1:1,4	54 ± 12 (16)	1 ± 1	7 ± 3	13 ± 5	0 ± 0
Focal lower limb	1 ± 1 (2)	1:2	52 ± 15 (6)	1 ± 1	1 ± 1	1 ± 2	0 ± 0
Focal axial	1 ± 1 (2)	1:1	62 ± 18 (3)	0 ± 0	2 ± 1	2 ± 1	8 ± 7
Segmental	33 ± 5 (63)	1:3,7	58 ± 12 (78)	4 ± 1	40 ± 6	99 ± 10	12 ± 12
Multifocal	6 ± 2 (11)	1:13	52 ± 14 (12)	3 ± 1	8 ± 3	14 ± 3	0 ± 0
Generalized	2 ± 1 (5)	1:2,5	49 ± 12 (6)	1 ± 1	6 ± 1	3 ± 1	0 ± 0

Average population over 20 years 2007–2016: 1580758. M:F = male: female -ratio. M:F was not counted for dystonia types with less than 10 patients.

The age in the day of diagnosis was calculated from the first visit with dystonia patients between 2007–2016. No significant age differences were seen between genders in any dystonia type. The age in the day of diagnosis differed significantly between CD or upper limb dystonia and blepharospasm (53 ± 11 and 51 ± 13 vs 64 ± 13, respectively) (p<0.005, Mann-Whitney U test, Table 1). No significant age difference was seen between focal and segmental dystonia. The statistical analysis was not done with lower cranial, lower limb, axial, multifocal and generalized dystonia due to small number of patients.

For upper limb dystonia, the highest prevalence was in age group 40–59 years, whereas for cervical and segmental dystonia the highest prevalence was in age group 60–79 years. The prevalence for focal upper cranial and laryngeal dystonia was highest in the oldest age group (Table 1). In the yearly prevalence comparison of all dystonia, the lowest prevalence was seen at the year 2007, rising to reach plateau 2014–2016 (S2 Fig). No patients with clear diurnal fluctuations were recorded.

## Discussion

The prevalence of dystonia in Finland has not been systematically studied previously. In our study, dystonia prevalence is based on analysis of national care register data and patient records. Present results suggest that the prevalence of CD in Finland (304 per million) is higher than in other countries with reported CD prevalence, including Norway, Iceland, England, Ireland, Germany, Italy, Serbia, Egypt, Japan, China, Thailand and Colombia (Table 2). In previous epidemiological studies the reported prevalence of dystonia in different countries has varied from 11 to 7320 per million [8, 9]. This may be partly due to the methodological differences and structural differences of health care systems [4]. In Finland, most patients are treated in the public health care system, and only a minor proportion uses private health care for dystonia. The high patient coverage in our study may explain the relatively high prevalence values (405 per million) compared with most previous studies (Table 2) [5, 8–25].

**Table 2:** The prevalence* per million persons and gender rates in previous dystonia studies.

Study	Country	All dystonia			Cervical dystonia		Blepharospasm		Upper Limb dystonia		Segmetal dystonia	
		Prevalence (n)	95% CI	M:F	Prevalence (n)	M:F	Prevalence (n)	M:F	Prevalence (n)	M:F	Prevalence (n)	M:F
Korczyn (1980)	Israel	11 (42)	7–13									
Nutt (1988)	USA	329 (34)		1,1	89 (5)		17 (1)		69£ (4)			
Kandil (1994)	Egypt	100 (4)	26–243		100 (4)							
Nakashima (1995)	Japan	61 (15)		1,1	29 (7)		16† (4)		16 (4)			
ESDE (2000)	Europe	152 (879)	142–162	1,6	57 (330)		36 (208)		14 (81)		32 (183)	
ESDE (2000)	Finland				233 (100)							
Castelon-Konkiewitz (2002)	Germany	142 (188)			54 (72)	1:1.3	31 (41)	1:2.2	3,8 ()		30 (39)	1:2.3
Muller (2002)	Italy, Tyrol	7320 (6)	319–1564									
Matsumoto (2003)	Japan	101 (147)		1:1.1	23 (34)		34 (49)		16 (23)		18 (26)	
Le (2003)	Norway	254 (129)		1:2.1	130 (66)	1:1.9	47 (24)	1:5.0	24 (12)	1:2.0		
Pekmezovic (2003)	Serbia	136 (165)	116–159	1:1.4	59 (72)	1:1.7	19 (23)	1:2.3	19 (23)	1:0.6	22 (27)	1:1.6
Butler (2004)	England	430 (43)	306–569									
Asgeirsson (2006)	Iceland	371 (107)	304–449	1:1.9	115 (33)	1:2.3	31 (9)	1:2.0	80£ (23)	1:1.9	31 (9)	1:2.0
Sugawara (2006)	Japan	151 (315)			28 (33)		104 (122)		11 (13)			
Jankovic (2007)	USA				3900# (248)							
Papantonio (2009)	Italy	127 (69)			45 (24)		68 (37)					
Bhidayasiri (2011)	Thailand	136 (141)	113–158	1:1.3	95 (99)	1:1.2	16 (12)	1:1.1	25 (21)	1:1.3		
Joensen (2016)	Faroe island	602 (29)	395–873	1:1.9	478 (23)				83 (4)			
Solano Atehortua (2016)	Colombia	712 (874)	488–937		248 (325)		105 (138)		104 (136)		100 (82)	
Wang (2016)	China	27 (1481)	26–28	1:2.0	8 (416)	1:1.9	12 (640)	1:2.2	0,6 (31)	1:0.7	5,5 (301)	1:2.5
Williams (2017)	Ireland	178 (592)	164–192		123 (410)	1:2.6	30 (102)	1:3.1	12 (39)	1:1.2		

*Isolated idiopathic or hereditary dystonia, if not otherwise stated.

† all cranial dystonia

# also non-isolated or acquired dystonia

£ all limb dystonia.

M:F male:female -ratio.

A report of dystonia prevalence in Finland was published previously as part of the Epidemiological Study of Dystonia in Europe epidemiology (ESDE) in 2000 [5]. Also then, the prevalence of CD in Finland (233 per million) was higher than in other studied countries. It was suspected to be artefactual due to the data collection method, which was partly through media, and the national care registry was not used [5]. The number of Finnish patients in the previous study was smaller in comparison with the present investigation, and the country-specific prevalence of other types of dystonia was not shown.

Two studies, which reported higher prevalence of CD, came from the USA and Faroe Islands, with the prevalence of 3900 and 478 per million, respectively [17, 21]. However, the study from the USA was based on an online survey with only 3% response rate, and included self-reported CD with non-isolated or acquired CD and without clinical validation [17]. The number of patients in the study from the genetically isolated Faroe Islands was small reflecting the size of the community [21]. Other dystonia prevalence studies excluding Finland reported prevalence of CD between 20 and 248 per million [5, 9, 10, 12–17, 19, 22, 23, 26] (Table 2). It is possible, that a positive selection bias occurs, when most CD patients in Finland are followed-up in specialized health care.

Regional variations in dystonia prevalence have been reported previously from other countries. In Japan and China, blepharospasm prevalence has been shown to exceed the prevalence of cervical CD [13, 23], while in western countries focal CD is the most common form of dystonia [4, 5]. Moreover, in southern parts of Europe, blepharospasm has been reported being more common than in northern parts. Williams et al. speculated that the higher proportion of blepharospasm in southern countries might be related to increased sun exposure [26]. Most countries with CD prevalence over 100 per million are Nordic countries with two exceptions of the USA and Colombia (Table 2). The higher prevalence in Finland might be linked to geographical factors or common genetic background, but again, the influence of methodological differences cannot be excluded.

The prevalence of other dystonia types did not differ considerably from other European studies (Table 2). Many existing studies have shown female predominance in focal dystonia, except for focal upper limb and axial dystonia [15, 16, 21, 23, 26]. The exact cause for such gender differences is not known, but it is hypothesized to be related to hormonal differences, gender-linked genetic susceptibility or exogenous factors [27]. The age in the onset of dystonia symptoms was not addressed in this study, but upper cranial and laryngeal dystonia had significantly higher age of diagnosis than CD or upper limb dystonia. The upper cranial and laryngeal dystonia prevalence was also the highest in the oldest age group. This may reflect the age of onset that has been shown to be higher in cranial dystonia than cervical or limb dystonia [12, 14, 16, 23, 26]. In our study, no significant gender-specific differences were observed within different age groups. In several studies the age of onset has been reported being younger in males with focal dystonia [13, 15, 20, 23, 26].

When comparing yearly prevalence in the years 2007–2016, the prevalence increased slightly until the year 2012. However, it is likely that this might merely be the reflection of study setting, and the lower prevalence is explainable by the lack of the follow-up visits of previously diagnosed dystonia patients in milder cases.

Our study has some limitations. It is registry and service-based study, so the data is based on patient and care records and no patients were contacted. The prevalence was measured as yearly prevalence for a period of ten years based on hospital visits with dystonia diagnosis. The medical records of 1522 patients with 1–2 visits because of dystonia were not available, thus the number of true prevalence might still be higher. On the other hand, it is likely that majority of these patients have incorrect diagnosis because they did not have any specialized health care visits because of dystonia and the percent of correct diagnosis with patients with 1–2 visits was small (17%). The patients whose surveillance was discontinued do not show in prevalence figures. The reasons for discontinuation were usually resolving of symptoms, no need for treatment, no suitable treatment available, or ineffective treatment.

Also, there is no data from the private sector which, however, represents only a minor fraction of the health care sector in Finland. Further, few patients with mild symptoms will not seek treatment [9, 17]. Most patients with generalized dystonia are missing from this study, because the onset of generalized dystonia occurs usually under the age of 20 years. Furthermore, some dystonia syndromes such as dopa-responsive dystonia and paroxysmal kinesigenic dystonia usually begin in childhood or adolescence, and therefore were not covered by this study.

In summary, adult onset focal CD was the most common type of dystonia with relatively high prevalence in Finland compared with other countries. The prevalence of other dystonia subtypes was comparable to other countries. The higher prevalence of CD might partially be explained by the better coverage of patients in the public health care system, but might also be attributable to geographical or genetic factors.

## Supporting information

S1 Fig The amounts of dystonia and non-dystonia patients with different number of ICD-10 G24 visits. (TIF)

S2 Fig The yearly average prevalence of different dystonia types. (TIF)
