# Supplementary figures and images for: The prevalence of adult-onset isolated dystonia in Finland 2007-2016
Source: PLoS One. 2018 Nov 20;13(11):e0207729. doi: 10.1371/journal.pone.0207729 (PMC6245745; doi:10.1371/journal.pone.0207729)

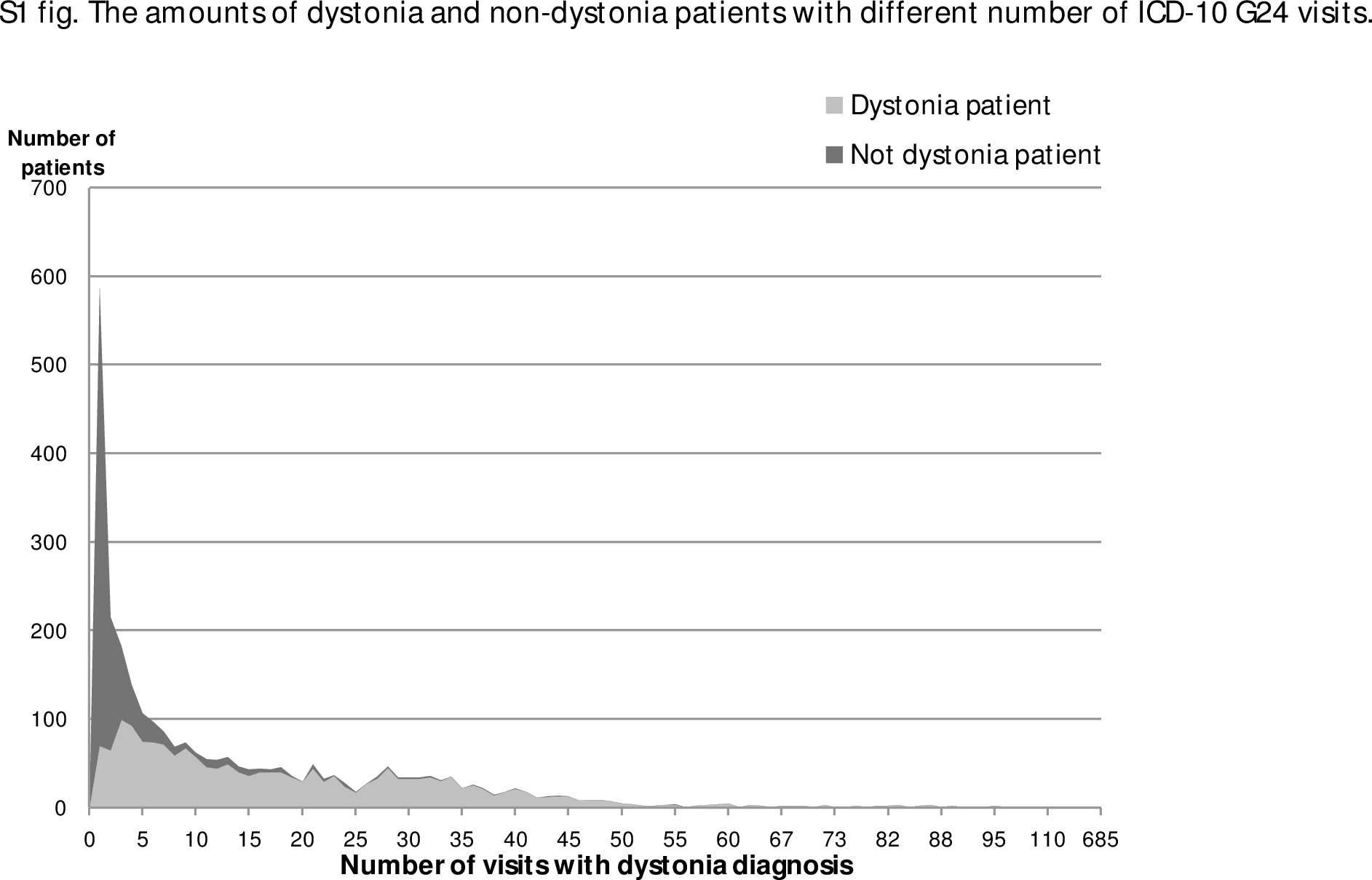

Supplement: S1 Fig — (TIF) [file pone.0207729.s001.tif]

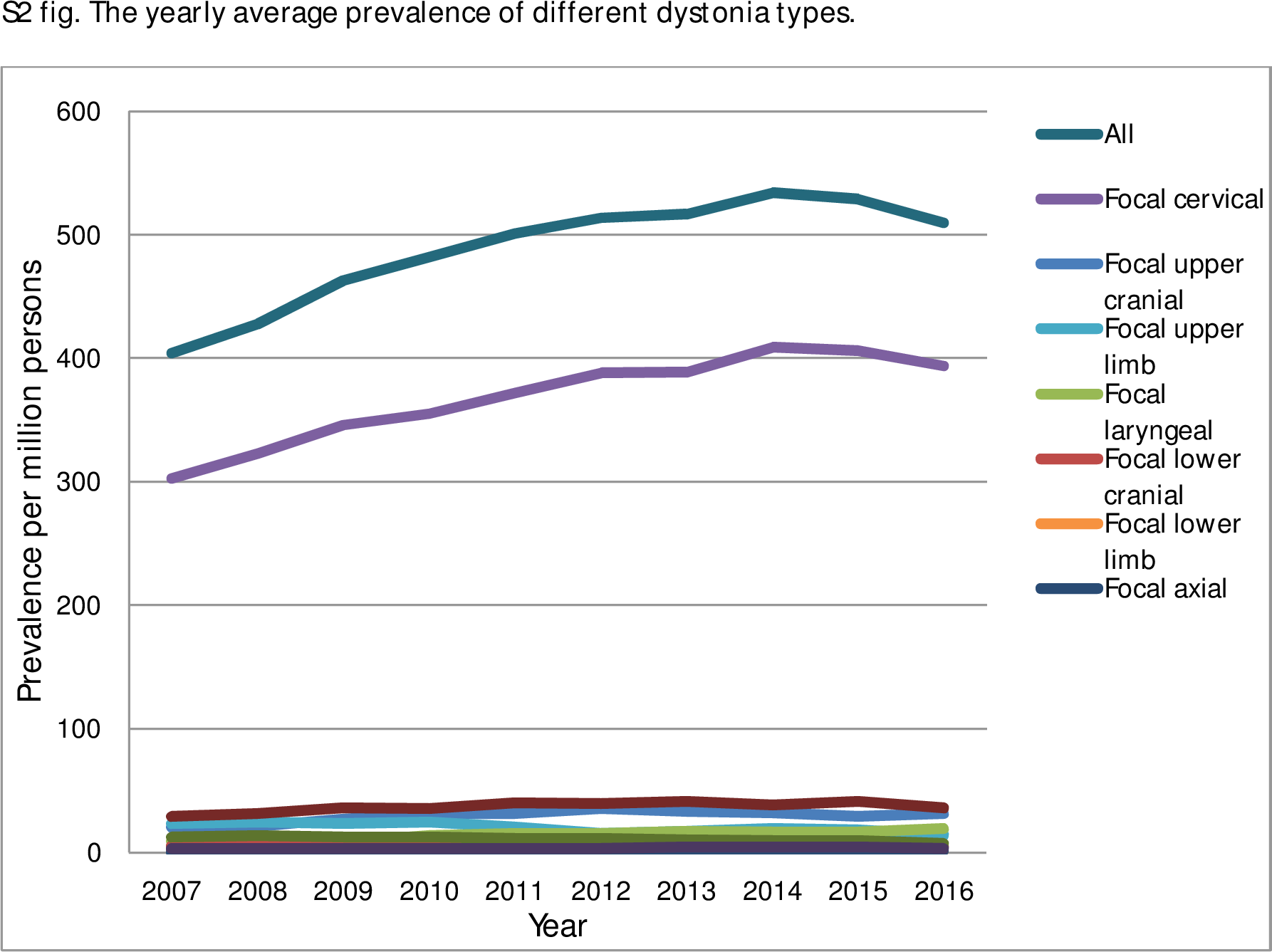

Supplement: S2 Fig — (TIF) [file pone.0207729.s002.tif]
